# Fibroblast *Has2* limits acute heart failure following myocardial infarction in male mice

**DOI:** 10.14814/phy2.70611

**Published:** 2025-11-18

**Authors:** Danielle T. Little, Kenneth R. Brittian, Caitlin Howard, Emma Pendergraft, Casey Colley, Ning Chen, Yu Yamaguchi, Richa Singhal, Joseph B. Moore, Marcin Wysoczynski, Yibing Nong, Steven P. Jones

**Affiliations:** ^1^ Center for Cardiometabolic Science, Christina Lee Brown Envirome Institute University of Louisville School of Medicine Louisville Kentucky USA; ^2^ Sanford Burnham Prebys Medical Discovery Institute La Jolla California USA

**Keywords:** extracellular matrix, fibrosis, heart failure, myocardial infarction

## Abstract

Changes to the extracellular matrix support acute wound healing following myocardial infarction. Fibroblasts regulate the composition of the extracellular matrix, in part, by secreting hyaluronan. Details surrounding the regulation, source, and impact of hyaluronan production after MI are largely unknown. We recently showed that activated fibroblasts produce hyaluronan via *Has2*; however, the extent to which this function impacts acute ventricular remodeling following myocardial infarction (MI) has not been tested. Hence, the goal of the present study was to elucidate the impact of fibroblast‐borne *Has2* expression in acute ventricular remodeling. Adult, male and female mice were subjected to non‐reperfused myocardial infarction and followed for 1 week and subjected to echocardiography and hearts were harvested for pathology and biochemical analyses. Mice were deficient in fibroblast‐derived *Has2* (*Has2* ^*−/−*^) or were littermate controls that were sufficient in fibroblast *Has2*. At 1‐week post‐MI, *Has2* ^*−/−*^ male mice had exacerbated heart failure reflected by lower cardiac output due to lower stroke volume, when compared to littermate males. The genotype effect was not evident in female mice. To assess potential mechanisms, we examined hearts for fibrosis, cardiomyocyte cross‐sectional area, and capillary density; there were no significant differences in any of these endpoints. Deletion of *Has2* also did not impact collagen organization, which could have indicated changes in ventricular stiffness. Fibroblast‐derived *Has2* supports cardiac function early after MI. The mechanism responsible for this and why it is not evident in female mice is unclear.

## 1 INTRODUCTION

Myocardial infarction (MI) activates cardiac fibroblasts, which participate in acute scar formation and remodeling (Frangogiannis, 2016). The function of cardiac fibroblasts in such replacement fibrosis is essential, and limitations to their function could impair the acute wound healing response in the heart (Daseke et al., 2020). Of the functions ascribed to fibroblasts, they are often most noted for their secretory capacity, which entails the secretion of an array of fibrillar collagen products. The deposition of collagen after MI is a central function that places fibroblasts as the foundational responders to form scar (Tallquist, 2020). Fibroblasts do, however, secrete many non‐collagenous factors (DeLeon‐Pennell et al., 2020). Yet, the role of many such factors remains to be examined.

Fibroblasts produce large amounts of glycosaminoglycans, such as hyaluronan. In fact, fibroblasts may produce even more hyaluronan than collagen (Green & Hamerman, 1964). Despite occupying such a conspicuous role in the secretome of fibroblasts, the role of hyaluronan in the heart is quite cryptic. Hyaluronan (Simpson et al., 2022) is often produced by the enzyme HAS2, which is encoded by *Has2* (in mice) (Kosaki et al., 1999). We have also shown that hyaluronan accumulates in and around the infarct territory following MI (Audam et al., 2025) and that activated fibroblasts per se produce copious amounts of HAS2‐derived high molecular weight hyaluronan in response to TGFβ (Little et al., 2025). Despite prior insights into whole‐body deletion of *Has2* in the context of myocardial ischemia/reperfusion injury (Petz et al., 2019), the role of fibroblast‐derived hyaluronan after MI has not, however, been defined.

Our present study was designed to test whether fibroblast‐derived hyaluronan was important in the early remodeling of the left ventricle after MI. We used fibroblast‐specific, inducible *Has2* deficient mice (Matsumoto et al., 2009). We assessed a battery of endpoints related to heart failure and ventricular remodeling. Our study provides primary evidence that fibroblast‐derived hyaluronan plays a key role in the acute phases of post‐MI heart failure. Such insights expand the portfolio of fibroblast functions, thereby generating a richer tapestry of the complicated events occurring following MI.

## 2 METHODS

All animals used in this study were on a C57BL6/J background. Male and female mice between the ages of 10–16 weeks were used in this study. All animals were used in compliance with the Guide for the Care and Use of Laboratory Animals, issued by the National Institutes of Health. The experimental protocols in this study have been reviewed and approved by the University of Louisville Institutional Animal Care and Use Committee. All mice were kept on a 12:12 light:dark cycle (lights on at 6:00 am, lights off at 6:00 pm). Mice were maintained in a barrier facility until baseline echocardiography. Breeders were fed breeder chow (LabDiet #5012); once weaned, mice were fed a standard rodent chow (LabDiet #5010). Mice were euthanized via anesthetic overdose, exsanguination, and heart removal.

### 2.1 Inducible, fibroblast‐specific *Has2*‐deficient mice

C57BL/6J‐background *Has2* floxed mice (*Has2* ^fl/fl^) have been described (Matsumoto et al., 2009). To create inducible fibroblast‐specific *Has2*‐deficient mice, we crossed *Has2* ^fl/fl^ mice with mutated estrogen receptor‐inducible, fibroblast‐specific Cre (Col1a2‐Cre) transgenic mice (The Jackson Laboratory, Stock No. 029567) to generate offspring heterozygous for the *Has2* allele and hemizygous for the Col1a2‐Cre transgene (*Has2* ^fl/+^::Col1a2‐Cre). Subsequently, these mice were crossed with homozygous *Has2* ^fl/fl^ animals to obtain *Has2* ^fl/fl^ homozygous, Col1a2‐Cre hemizygous mice (*Has2* ^fl/fl^::Col1a2‐Cre). *Has2* ^fl/fl^ littermates lacking the tamoxifen‐inducible Cre transgene were used as controls. Transnetyx is the commercial service we used for genotyping.

### 2.2 Tamoxifen preparation and administration

A tamoxifen stock solution at a concentration of 20 mg/mL was created by dissolving 200 mg of tamoxifen (Sigma‐Aldrich, Cat. #T5648) in 1 mL of 200‐proof ethanol. The volume was then adjusted to 10 mL using peanut oil, and the mixture was incubated at 37°C with vortexing. Any remaining tamoxifen not in solution was aided to dissolve through brief sonication. Fibroblast‐specific ablation of *Has2* was induced via daily intraperitoneal (i.p.) injection of tamoxifen (80 mg/kg) for five consecutive days in homozygous floxed, Cre positive and Cre negative littermates 5 days prior to MI—denoted from this period forward as *Has2* ^*+l+*^ and *Has2* ^*−/−*^, respectively. Echocardiograms were collected 7 days post‐MI.

### 2.3 Myocardial infarction

*Has2* ^*−/−*^ mice (12–16 week‐old) and their wild‐type littermates (*Has2* ^*fl/fl*^) were subjected to non‐reperfused myocardial infarction (MI) as described previously (Audam et al., 2020; Brainard et al., 2013; Sansbury et al., 2014; Watson et al., 2010; Wysoczynski et al., 2016). Briefly, mice were anesthetized with intraperitoneal injections of ketamine hydrochloride (50 mg/kg) and sodium pentobarbital (50 mg/kg). Mice were orally intubated and ventilated; the ventilator's room‐airport was supplemented with oxygen. A 7–0 silk suture was passed under the left coronary artery and tied. The chest and skin were closed in layers. Mice were extubated upon recovery of spontaneous breathing. Analgesia (ketoprofen, 5 mg/kg) was provided prior to surgery and by 24 and 48 h post‐surgery. The surgeon was blinded to mouse genotype. Seven days after MI, all mice were subjected to an echocardiogram to confirm sufficient depression of cardiac function (LVEF <40%). We excluded 11 *Has2* ^*+l+*^ and 9 *Has2* ^*−l−*^ mice.

### 2.4 Echocardiography

Transthoracic echocardiography of the left ventricle was performed as described (Audam et al., 2020; Audam et al., 2021; Brainard et al., 2013; Sansbury et al., 2014; Wang et al., 2013) with adjustments for the VisualSonics Vevo 3100 system. The sonographer was blinded to the treatment group. The parasternal long axis (PSLAX) view, in M‐Mode, was used to obtain two‐dimensional images for the measurement of the thickness of the left ventricle anterior wall (LVAW), the left ventricular interior diameter (LVID), and the left ventricle posterior wall (LVPW). Fractional shortening (FS) was calculated by using the formula [(LVID; d − LVID; s)/LVID; d] × 100. Four measurements were taken and averaged for each parameter. The parasternal long axis (PSLAX) view, in B‐Mode, was used to obtain two‐dimensional images for the measurement of heart rate, with six measurements taken and averaged. The parasternal short axis (PSAX) view, using Simpson's method, was used to obtain two‐dimensional images for the measurements of LV end‐diastolic volume (LVEDV), LV end‐systolic volume (LVESV), LV stroke volume (SV), LV ejection fraction (EF), and cardiac output (CO), with five measurements taken and averaged for each parameter. Pulse wave (PW) Doppler in apical four chamber view was used to obtain two‐dimensional images for the measurement of the isovolumic relaxation time (IVRT), with nine measurements taken and averaged.

### 2.5 Pathology

Following final echocardiography, hearts were excised and arrested in diastole with 2% KCl. Hearts were then sectioned into 1‐mm short‐axis sections. All sections were fixed with 10% formalin; the samples were subsequently embedded, cut, and mounted. MATLAB version 9.7.01216025 (R2019b) was used to compile data files exported from the Keyence analysis software to generate a summary file for data analysis, and the microscopist was blinded to group assignment. Transverse heart sections from 1‐week post‐MI mice were fixed in neutral‐buffered 10% formalin and embedded in paraffin wax. The hearts were sliced into 4 μm sections, deparaffinized in an oven, dehydrated in xylene, and rehydrated with ethanol and water. All stained tissue slides were imaged with a Keyence BZ‐X810 microscope and analysis was done with the Keyence software coupled with customized Matlab macros.

### 2.6 Quantification of scar size by picrosirius red staining

To visualize fibrosis accumulation (Audam et al., 2020, 2021, 2025; Brooks et al., 2015; Fischer et al., 2024; Little et al., 2025; Stephan et al., 2025; Watson et al., 2014), heart sections were stained with Picrosirius Red made from Direct Red 80 (Sigma‐Aldrich 365,548‐5G) and saturated Picric Acid (Sigma‐Aldrich P6744‐1GA). Formalin‐fixed, paraffin‐embedded myocardial tissue sections were heated at 75°C for 30 min, deparaffinized in xylene, and stepwise rehydrated through incubation in decreasing concentrations of ethanol (100%, 96%, 90%, 80%) before being placed in deionized water. Picrosirius red stain was prepared using 0.1% (w/v) Direct Red 80 (365548‐5 G; Sigma‐Aldrich) in picric acid (P6744–1GA; Sigma‐Aldrich). The sections were incubated in Picrosirius red solution for 1 h, washed twice in 0.5% acetic acid (1 min each), stepwise dehydrated with ethanol, placed in xylene, and then mounted under glass coverslips using Permount Mounting Media (Electron Microscopy Sciences, Cat. #17986‐05). Slides were imaged under automation at 20× magnification using a Keyence BZ‐X810 microscope. Morphometric parameters, such as infarct width, were manually measured via ImageJ in midpapillary level sections. Furthermore, the analysis software supplied by Keyence was employed to ascertain the fibrotic area for each myocardial segment. Subsequently, total scar size (mass in mg) was enumerated by the summation of scar masses corresponding to each transverse myocardial segment (apex to base; 4–5 segments per heart): scar mass (mg) = [section mass (mg)] × [(scar area (mm^2^))/(section area (mm^2^))]. The right ventricle was removed from the image, and the total fibrosis percentage was quantified in the remote zone, infarct zone, and left ventricle.

### 2.7 Quantification of hyaluronan area by HABP staining

To examine HA accumulation (Audam et al., 2025; Little et al., 2025), formalin‐fixed, paraffin‐embedded myocardial tissue sections were heated at 70°C for 30 min, deparaffinized in xylene, and stepwise rehydrated through incubation in decreasing concentrations of ethanol (100%, 95%, 70%, 50%, and 0%). Antigen retrieval was performed by incubating the slides in TrypLE Express (Thermo 12604‐021) in a 37°C water bath for 15 min. The slides were then washed in 1× DPBS (Sigma D5652) (3 times for 5 min) and blocked with avidin (Thermo 004303) for 15 min before being washed in 1× DPBS (3 times for 3 min). Subsequently, the slides were blocked with biotin (Thermo 004303) for 15 min. The slides were then incubated in 10% FBS for 1 h at room temperature before incubating in biotinylated‐HABP (SIGMA 385911) (0.5 μg/μL) diluted to 2:298 in 10% FBS overnight at 4°C. Slides were washed in 1× DPBS (3 times for 3 min) and incubated in 0.7 μL Alexa Fluor 568 (Thermo S11226) diluted in 199.3 μL 10% FBS for 30 min at room temperature. Subsequent washes with 1× DPBS (2 times for 3 min) followed before the sections were counterstained with 1 μg/mL DAPI for 15 min at room temperature to visualize the nuclei. Sections were washed with 1× DPBS (3 times for 3 min) and incubated in Sudan Black solution (350 mL 100% ethanol, 500 mg Sudan Black B (Sigma‐Aldrich, Cat. #199664) to 500 mL with deionized water) for 30 min at room temperature. After washing in 1× DPBS (3 times for 5 min), the sections were mounted using PermaFluor Aqueous Mounting Medium (Fisher Scientific, Cat. #TA030FM). For imaging, the entire heart section was captured at 20× magnification using a Keyence BZ‐X810 microscope. HA percentage was quantified using the same methods above.

### 2.8 Determination of cardiomyocyte cross‐sectional area

Cardiomyocyte hypertrophy was assessed (Audam et al., 2020, 2021; Brooks et al., 2015; Facundo et al., 2012; Fischer et al., 2024; Sadri et al., 2022; Stephan et al., 2025; Watson et al., 2014). The borders of the cardiomyocytes were stained with wheat germ agglutinin (WGA CF™ 488A conjugate, Biotium) and the capillaries with Isolectin B4 DyLight 649 (Vector Labs DL‐1208‐.5) in 10 mM HEPES solution with calcium chloride dihydrate. DAPI was used to visualize the nuclei. Only myocytes with centrally located nuclei and a circularity of 50–100 were used for quantification. As above, formalin‐fixed, paraffin‐embedded myocardial tissue sections were heated at 70°C for 30 min, deparaffinized in xylene, and stepwise rehydrated through incubation in decreasing concentrations of ethanol (100%, 96%, 90%, 80%, and 0%). Antigen retrieval was performed by immersing the slides in citrate buffer (2.4 g/L sodium citrate tribasic dehydrate, 0.35 g/L citric acid, pH 6.0) and boiling for 15 min. Subsequently, the slide container was cooled on ice. After three 3‐min washes with 1× DPBS, the sections underwent incubation with Wheat Germ Agglutinin‐Rhodamine (WGA) (Vector Laboratories, Cat. #RL‐1022) (1:50 dilution) for 30 min at room temperature. Subsequent washes with 1× DPBS (3 times, 3 min each) followed. To visualize nuclei, the sections were counterstained with 1 μg/mL DAPI for 10 min at room temperature and then washed with 1× DPBS (3 times, 3 min each). To minimize autofluorescence signal, the sections underwent a 15‐min incubation in Sudan Black solution (350 mL 100% ethanol, 500 mg Sudan Black B from Sigma‐Aldrich, Cat. #199664, q.s. to 500 mL with deionized water) at room temperature. After washing in 1× DPBS (5 times, 3 min each) and deionized H_2_O (3 min), the sections were mounted using PermaFluor Aqueous Mounting Medium (Fisher Scientific, Cat. #TA030FM). For imaging, the entire heart section was captured at 20× magnification using a Keyence BZ‐X810 microscope, ensuring an unbiased representation of the entire sample. Automated analysis of cardiomyocyte cross‐sectional areas within images was performed using Keyence analysis software.

### 2.9 Determination of capillary density

Capillary density was assessed (Audam et al., 2020, 2021). Myocardial tissue sections were deparaffinized and rehydrated as above. The sections were then incubated in Wheat Germ Agglutinin‐Rhodamine (WGA; Vector Laboratories, Cat. #RL‐1022; 1:50 dilution) for 30 min at 37°C. After three 3‐min washes with 1X DPBS, the sections incubated in *Griffonia simplicifolia* Lectin I (GSL I) Isolectin‐B4‐Fluorescein (Vector Laboratories, Cat. #FL‐1201–0.5) diluted 1:25 in isolectin staining solution (1 mM CaCl_2_ and 10 mM HEPES in 1× PBS) for 1 h at room temperature followed by three 3 min washes in 1× DPBS. The sections were counterstained with 1 μg/mL DAPI for 15 min at room temperature to visualize nuclei and then washed with 1× DPBS (3 times, 3 min each). Sections were incubated in Sudan Black solution (350 mL 100% ethanol, 500 mg Sudan Black B from Sigma‐Aldrich, Cat. #19966, to 500 mL with deionized water) at room temperature for 30 min. Sections were washed and mounted as above. Images were acquired at 20× magnification using a Keyence BZ‐X810 microscope. To quantify capillary density, automated analysis of cardiomyocyte cross‐sectional areas within images was performed using Keyence analysis software. Capillary density was calculated by dividing the number of capillaries by the total tissue area in the image (mm^2^).

### 2.10 Reverse transcriptase PCR and real‐time PCR

Total cardiac RNA was extracted and used to make cDNA. The relative levels of mRNA transcripts were quantified by real‐time PCR using Power SYBR Green (Thermo Fisher Scientific) on a real‐time PCR system (QuantStudio5). Most primers were made using NCBI Primer Blast except HPRT primers (PPM03559E‐200, QIAGEN). The data were normalized to mouse HPRT mRNA threshold cycle (CT) values by using the ΔΔCT comparative method. All primer sequences are as reported (Little et al., 2025) and are as follows: (Has1 Forward: GAGGCCTGGTACAACCAAAAG; Has1 Reverse: CTCAACCAACGAAGGAAGGAG; Has2 Forward: GCCTCGCATCTCATCATC; Has2 Reverse: CCTGCCCATAACTTCGCTGA; Has3 Forward: TGGACCCAGCCTGCACCATTG; Has3 Reverse: CCCGCTCCACGTTGAAAGCCAT).

### 2.11 Hyaluronan ELISA

Whole heart tissue from both *Has2* ^*fl/fl*^ and *Has2* ^*−/−*^ with 1 week. MI was weighed and homogenized in water using a Next Advance Bullet Blender. A concentration of 0.14 mg/μL was taken out of stock homogenate using Cell Lysis Buffer 2 (R&D Systems: 895347), and tissue was lysed for 30 min at room temperature with gentle agitation. Samples were centrifuged to remove debris. Samples were diluted to a 1:500 dilution. Quantikine ELISA: Hyaluronan Immunoassay (R&D Systems: DHYAL0) was used to assess HA concentration in each sample as in previous methods (Audam et al., 2025; Little et al., 2025).

### 2.12 Hydroxyproline assay

Whole heart tissue from both *Has2* ^*fl/fl*^ and *Has2* ^*−/−*^ at 1 week post‐MI was weighed and homogenized in water using a Next Advance Bullet Blender. A concentration of 0.1 mg/μL was taken out of stock homogenate and hydrolyzed using 12 N NaOH and boiled for 1 h at 120°C. Hydrolyzed sample was neutralized using 12 N HCl and hydroxyproline concentration in samples was observed using the Hydroxyproline Assay (Abcam Ab222941), as we previously reported (Little et al., 2025).

### 2.13 Hyaluronan isolation, sizing, and analysis

Hyaluronan was isolated from cultured media and heart tissue as described (Little et al., 2025).

### 2.14 Second harmonic generation microscopy

As previously described (Fischer et al., 2024), myocardial sections obtained from 1‐week post‐myocardial‐infarcted mice were deparaffinized by heating at 80°C for 30 min and placing in xylene. Sections were stepwise rehydrated in ethanol (100%, 96%, 90%, 80%) and then placed in deionized water for 3 min. Sections were finally placed in 1× PBS and then subjected to second harmonic generation (SHG) imaging using a Nikon A1R MP multiphoton microscope with an Apo LWD 25× water immersion objective. The excitation laser was tuned to 920 nm and the SHG signal was collected through a DAPI bandpass filter with an emission wavelength of 446 nm. Whole‐heart cross‐section images were acquired at a resolution of 0.34 μm per pixel and a scan speed of 1.9 frames per second. Six regions of interest (ROI: 450 × 450 pixels) per SHG‐imaged heart, focusing on the longitudinal alignment of collagen fibers, were selected. All ROIs underwent computational analysis using MATLAB software framework packages, CurveAlign and CT‐FIRE, as we performed previously (Fischer et al., 2024), enabling the quantitative evaluation of macrostructural attributes of the collagen network (i.e., fiber alignment, straightness, and width).

### 2.15 Flow cytometry

Peripheral blood, bone marrow, and spleen samples were collected and used for flow cytometry analysis (BD Fortessa) of basic immune cell populations. Prior to staining, a small aliquot was taken from each sample for cell counting. All samples were stained on ice for 20 min. At this point, a red blood cell lysis buffer (BD lysis buffer, #555899) was used to remove red blood cells from the peripheral blood samples. All samples were washed and resuspended in PBS for analysis via flow cytometry. A CD11b antibody was first used to separate lymphocytes (CD11b^−^) and non‐lymphocytes (CD11b^−^). Within the CD11b^−^ population, CD3 and B220 expression were used to differentiate between T cells and B cells, respectively. Cells that were CD11b^+^Ly6G^+^ were identified as neutrophils. Within the CD11b^+^Ly6G^−^ cells, CD115 expression was used to separate monocytes (CD115^+^) and dendritic cells (CD115^−^). In peripheral blood samples, CD11b^+^Ly6G^−^SSC^High^ cells were identified as eosinophils. Antibodies and fluorochromes used are listed in Table S1. All flow analysis was completed using FlowJo, using gating strategies as shown in Figure S1. If 100,000 flow events were not collected for a sample, that sample was excluded. The percent that each population made up of the total events collected was multiplied by the cell count of each sample to generate an estimated number of cells per population per sample.

### 2.16 Statistical analysis

Statistical analyses were conducted using GraphPad Prism version 10 for Mac (GraphPad Software, La Jolla, California, USA; http://www.graphpad.com). Detailed statistical procedures for each experiment are provided in the corresponding figure legends. Statistical significance was defined as *p*‐values less than 0.05 in all cases. Data supporting this study can be made available upon reasonable request.

## 3 RESULTS

### 3.1 Confirmation of *Has2* deletion

**FIGURE 1:**
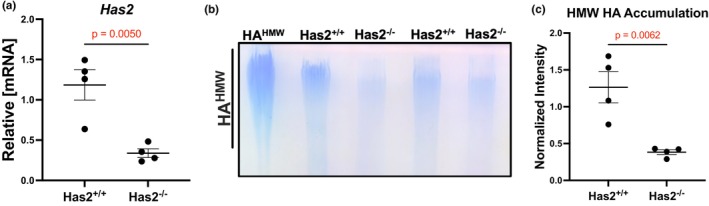
Confirmation of fibroblasts specific Has2 deletion in mice. (a) Relative mRNA expression of *Has2* is significantly decreased in fibroblasts isolated from *Has2* ^*−/−*^ mice compared to *Has2* ^*+/+*^ mice. (*n* = 4) (b) Representative gel showing relative size and abundance of hyaluronan. High molecular weight hyaluronan (HA^*HMW*^) was loaded as a molecular weight marker/positive control. (c) Quantification of stained agarose gels. HA is significantly decreased in *Has2* deleted mice (*Has2* ^*−/−*^) compared to control. (*n* = 4).

To confirm *Has2* was deleted, we isolated cardiac fibroblasts from tamoxifen‐treated, Col1a2‐Cre positive and negative mice (all mice were homo‐floxed for *Has2*). From the fibroblast cultures, we isolated RNA from the cells and HA from the media. Our data indicate that the *Has2* mRNA was significantly decreased in the *Has2* ^*−/−*^ fibroblasts compared to *Has2* ^*fl/fl*^ fibroblasts (Figure 1a). We also noted a significant decrease in the level of high‐molecular weight HA (HA^HMW^) accumulation in the *Has2* ^*−/−*^ fibroblast media compared to *Has2* ^*+/+*^ media (Figure 1b,c). Examining the mRNA expression of *Has1* and *Has3*, we found a significant increase in *Has1* levels in Has2^−/−^ mice compared to Has2^+/+^ mice (Figure S2A), suggesting that *Has1* may be compensating for the loss of *Has2*; however, despite this upregulation, HA^HMW^ levels remained significantly reduced post‐*Has2* deletion, indicating that the deletion was effective. In contrast, *Has3* mRNA expression remained unchanged following *Has2* deletion (Figure S2B).

### 3.2 Cardiac function 7 days post‐MI

**FIGURE 2:**
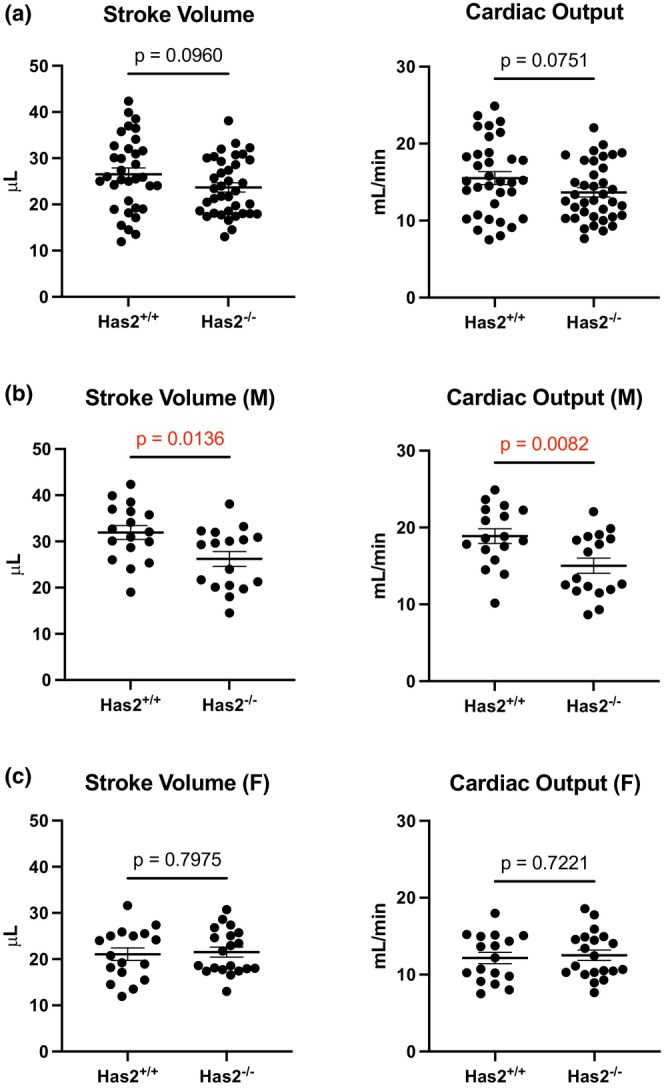
*Deletion of fibroblast‐specific Has2 causes reduced cardiac function in male mice*. (a) Stroke volume and cardiac output. (b) Stroke volume and cardiac output in male mice. (c) Stroke volume and cardiac output in female mice. *Has2* ^*+/+*^ *n* = 34 (17F, 17M) versus 7 days post‐MI *Has2* ^*−/−*^ *n* = 37 (20F, 17M).

To determine the impact of fibroblast *Has2* deletion in acute remodeling, we subjected mice to MI and performed echocardiography at 7 days post‐MI. In males, the most striking finding was the exacerbation of heart failure—that is, both stroke volume and cardiac output were significantly lower in *Has2* ^*−/−*^ mice compared to *Has2* ^*fl/fl*^ mice (Figure 2b). In aggregated data (i.e., both sexes), we also observed a significant increase in the IVRT in *Has2* ^*−/−*^ mice, but no differences when disaggregated by sex (Table S2). Other indices were not different between *Has2* ^*−/−*^ and *Has2* ^*fl/fl*^ mice, regardless of sex. Hence, fibroblast‐derived hyaluronan is at least partially pro‐adaptive during acute post‐MI remodeling in male mice.

### 3.3 Hyaluronan accumulation in the heart

Hyaluronan and collagen accumulate in the infarct (Little et al., 2025). To determine if deletion of fibroblast *Has2* causes reduction of the HA seen in the infarct zone we performed an Hyaluronan Binding Protein (HABP) stain on heart tissue slices taken from both *Has2* ^*−/−*^ and *Has2* ^*fl/fl*^ mice. To measure overall HA amount in the whole heart, we homogenized whole hearts and used an HA ELISA assay to measure HA concentration. When compared to *Has2* ^*fl/fl*^, *Has2* ^*−/−*^ showed no significant difference in the HA concentration in the total heart (Figure S3). When observing the HA accumulated by fibroblasts isolated from infarcted cardiac tissue, we no longer saw a significant reduction in *Has2* ^*−/−*^ *mice*. Additionally, *Has2* gene expression was not significantly altered after MI (Figure S4). Interestingly, while *Has1* expression was elevated prior to infarction, this increase was no longer present 7 days post‐MI, whereas *Has3* expression remained unchanged (Figure S4).

### 3.4 Scar size and collagen alignment

**FIGURE 3:**
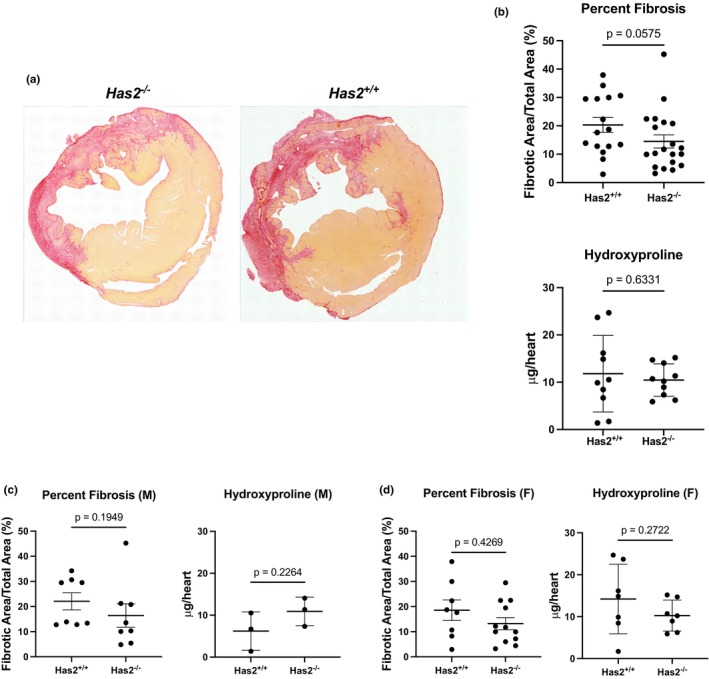
Deletion of Has2 in cardiac fibroblasts 7 days post‐MI does not induce fibrosis. (a) Representative images taken after staining with Picrosirius red which stains collagen. (b) Quantification of tissue stained with Picrosirius red (PSR) and quantification of hydroxyproline assay. (c) Males (d) Females PSR: Unpaired *t*‐test with Mann–Whitney test. *Has2* ^*+/+*^ *n* = 16 (8F, 8M), *Has2* ^*−/−*^ *n* = 20 (8F, 12M). Hydroxyproline: Unpaired *t*‐test. *Has2* ^*+/+*^ *n* = 10 (7F, 3M), *Has2* ^*−/−*^ *n* = 10 (7F, 3M).

**FIGURE 4:**
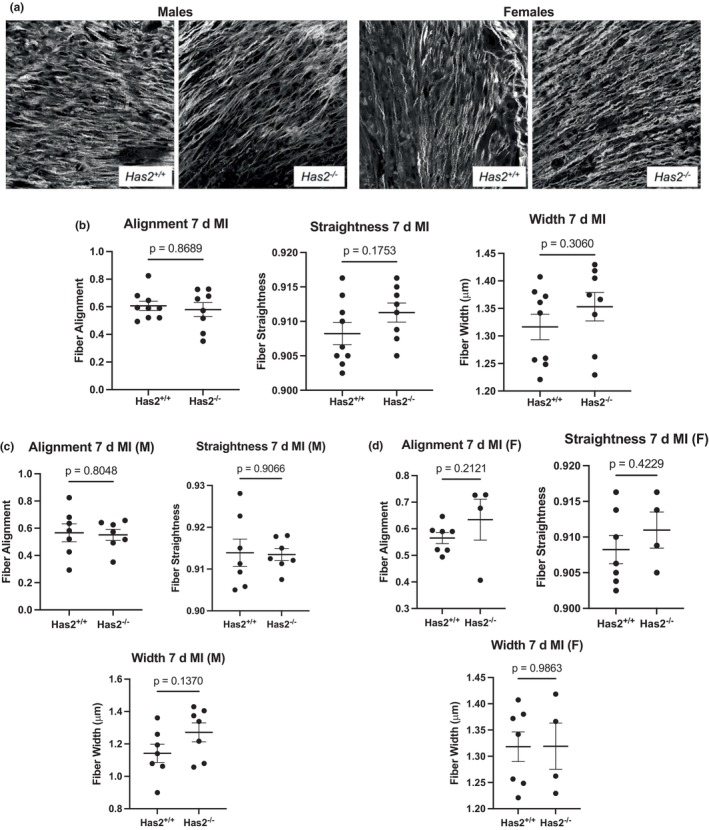
Deletion in fibroblast‐specific Has2 did not cause changes in structural and organizational properties of collagen fibers. (a) Representative second‐harmonic generation (SHG) microscopy images depicting infarct scar regions in *Has2* ^*+/+*^ and *Has2* ^*−/−*^ mice. Images are of high resolution (4506 × 4506 pixels) and were taken 1 week after permanent ligation. (b) Graphs illustrating mean fibrillar collagen alignment, straightness, and width ± SEM in *Has2* ^*+/+*^ (*n* = 9) and *Has2* ^*−/−*^ (*n* = 8) animals 7 days post MI. Data are representative of analyses performed on six distinct ROIs. (c) Males. (d) Females. Statistical procedures: Data normality was assessed using the Shapiro–Wilk test. Alignment data were analyzed using the non‐parametric Mann–Whitney *U* test. Width and Straightness data were analyzed by an unpaired, two‐tailed Student's *t*‐test.

We used Picrosirius red to estimate scar size. There was no difference in fibrosis in *Has2* ^*−/−*^ compared to *Has2* ^*+/+*^ hearts 7 days post‐MI (Figure 3a). Disaggregation of the data by sex also indicated no significant differences (Figure 3b,c). Additionally, we measured collagen content using a hydroxyproline assay and observed no changes in total collagen levels in the heart following cardiac fibroblast‐specific *Has2* deletion 7 days post‐MI (Figure 3a–c). To assess collagen architecture, we used Second Harmonic Generation microscopy to observe the collagen alignment, straightness, and width. We found no significant differences between *Has2* ^−/−^ and *Has2* ^+/+^ mice (Figure 4b). Additionally, there were no sex‐dependent changes in collagen fiber alignment, straightness, or width (Figure 4c,d).

### 3.5 Hypertrophy and vascular density

**FIGURE 5:**
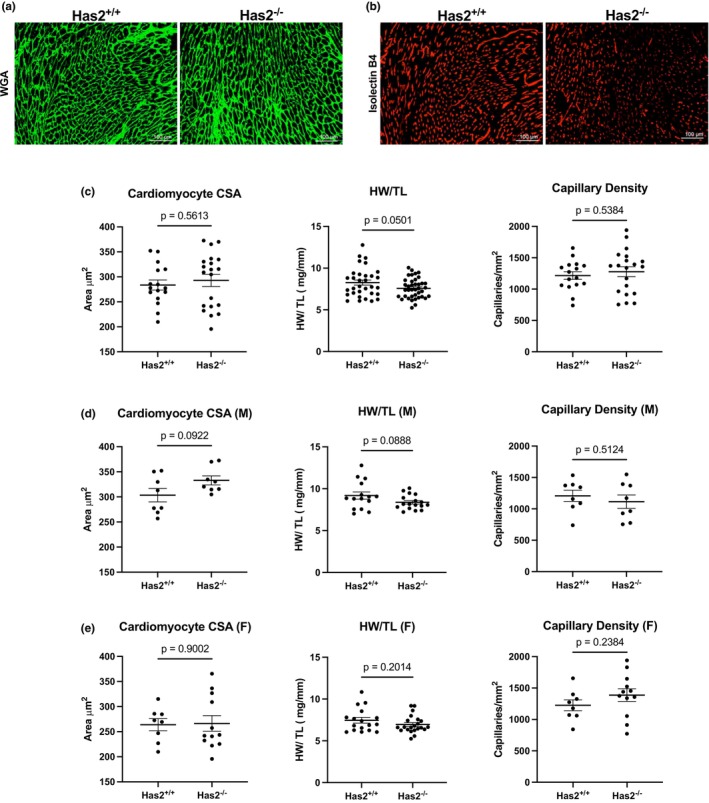
Fibroblast‐specific Has2 deletion does not cause changes in cardiomyocyte hypertrophy in mice 7 days post‐MI. We assessed the impact of *Has2* deletion on cardiomyocyte size by wheat germ agglutinin (WGA) and measuring heart weights. (a) WGA‐stained (red) *Has2* ^*+/+*^ and *Has2* ^*−/−*^ hearts. (b) Isolectin B4‐stained (green) hearts to determine vascular density. (c) Quantification of WGA‐stained *Has2* ^*+/+*^ (*n* = 14; 7M, 7F) and *Has2* ^*−/−*^ (*n* = 19; 7M, 12F) hearts. Heart weight/tibia length ratio for *Has2* ^*+/+*^ (*n* = 30; 15M, 16F) and *Has2* ^*−/−*^ (*n* = 39; 17M, 22F) hearts. Quantification of vascular density in isolectin B4‐stained *Has2* ^*+/+*^ (*n* = 16; 8M, 8F) and *Has2* ^*−/−*^ (*n* = 20; 8M, 12F) hearts. (d) Males (e) Females.

To evaluate the impact of fibroblast‐specific *Has2* deletion on cardiac hypertrophy and vascular density, we performed wheat germ agglutinin (WGA) staining (Figure 5a) and isolectin B4 staining (Figure 5b) 7 days post‐MI. Neither cardiac hypertrophy nor vascular density showed significant changes overall due to *Has2* deletion (Figure 5c). Additionally, no sex‐dependent differences were observed in hypertrophy or vascular density (Figure 5d,e).

### 3.6 Hematology

No significant changes were observed in the bone marrow (cells/tibia) or spleen (cells/mg tissue) samples in any of the identified populations. We observed a significant increase in circulating T cells and neutrophils (cells/μL blood) in the female fibroblast specific *Has2* ^*−/−*^ mice. This same increase was not observed in the peripheral blood samples collected from male mice suggesting a potential sex dependent effect of fibroblast derived hyaluronan. These data are provided as additional characterization. Intentional studies would need to be completed to better explore the implications of changes in neutrophils and T cells (Table S3), and to understand whether there are changes in the heart.

## 4 DISCUSSION

We addressed the role of fibroblast‐derived *Has2* in acute ventricular remodeling post‐MI. Deletion of *Has2* exacerbated heart failure in male mice, as evidenced by reduced cardiac output. Despite the reduction in cardiac output post‐MI, there were no differences in cardiomyocyte hypertrophy or capillary density. There was no difference in bulk fibrosis, and collagen fiber organization was not different in *Has2* ^*−/−*^ hearts after MI. These data underscore an essential role for fibroblast‐derived *Has2* in acute post‐MI ventricular remodeling.

A recent study (Petz et al., 2019) showed that induced whole‐body deletion of *Has2* in adult mice leads to increased scar size 3 weeks after ischemia–reperfusion (I/R) injury, accompanied by a reduced ejection fraction compared to controls. There are several differences between the previous study (Petz et al., 2019) and our present data. Our approach used an inducible, fibroblast‐enriched Cre‐driver, essentially restricting deletion to the fibroblasts, whereas the prior approach (Petz et al., 2019) used whole‐body deletion. HA serves various roles in different tissue beds, and some of those roles are not known. Our use of a fibroblast‐specific *Has2* ^*−/−*^ mouse supports our contention that fibroblasts are a key source of HA following MI, and their production of HA is essential. Another difference between the prior study (Petz et al., 2019) and ours is their use of a non‐reperfused model. First and foremost, the possibility that acute differences in infarct size drove the results is a likely explanation. When assessed at 3 weeks post‐myocardial ischemia/reperfusion (MI/R), scars were significantly larger in the *Has2* deficient mice. Data (i.e., late gadolinium enhancement cMRI) were provided to discount the notion of acute differences in infarct size; however, direct assessment of infarct size and area‐at‐risk was not performed. Thus, deleting *Has2* in multiple tissues, then subjecting mice to MI/R, may have resulted in larger initial infarct sizes in *Has2* deficient mice. In our present study, we wanted to limit the possibility of differences in infarct size and use a severe model of heart failure. Our non‐reperfused MI model eliminates group differences in infarct size (Lindsey et al., 2021).

Fibroblasts are a major source of HA, particularly HA^HMW^. We recently showed that HA^HMW^ suppresses macrophage phagocytosis and efferocytosis, which could contribute to prolonged inflammation (Audam et al., 2025). In the context of the present findings, our prior work might be difficult to contextualize. Here are our thoughts. In the acute post‐MI setting, reducing the capacity to produce HA caused impaired cardiac output (in males). The reason for this is not known, though we speculate that it could be because losing HA unfavorably altered the organization of the matrix and perhaps had no impact on the immune response. It is also possible and untested that HA could have had a favorable impact on cardiomyocyte function, and whether such an effect was through HA receptors and/or supplementing carbon flux through intermediary metabolism is worthy of experimental pursuit. Beyond the present study design, that is, in the chronic condition (not yet tested), we think the persistent production of HA could impact inflammation in that it would support/prolong non‐resolving inflammation and contribute to pathology. Indeed, there are many intriguing possibilities to test.

So, why is there a change in stroke volume but no other histological aspects of ventricular remodeling? In other words, what is the mechanism? This is an excellent question that we cannot, at present, answer but are pursuing. Changes in preload and/or afterload could impact stroke volume. There are several factors that could impact diastolic filling, though one might expect a significant reduction in end diastolic volume. It is, of course, also possible that changes to the stiffness of the myocardium could impair filling and ejection, which may or may not impact diastolic or ventricular volumes. Such a scenario would be consistent with our findings. Further studies would be required to elucidate the explanation. Regardless, the present data are reflective of consensus statements on the definition of heart failure (Heidenreich et al., 2022).

In terms of the regulation of the expression of *Has* family genes, there is an interesting point to note. In cardiac fibroblasts, deletion of *Has2* caused an increase in *Has1*, but not *Has3*, mRNA. This invites speculation of the oft‐queried “compensatory increase”. Yet, despite this interesting finding, there was no notable impact on HA production. So, although molecular intrigue exists and warrants pursuit by others, this increase in *Has1* did not acutely compensate for the loss of *Has2*. It is, nevertheless, possible that the increase in *Has1* had a cryptic impact that was independent of HA. Such wild‐eyed speculation is just that for now; however, it should be noted that much about the production, degradation, and regulation of HA is not known, so we should be open to possibilities.

There are several aspects of our study design to acknowledge. This Cre driver has been used in many MI studies. Hence, there is little reason to believe the Cre driver per se is responsible for the observed results. Moreover, we have published that Cre activation (of the present Cre driver) with tamoxifen causes no sustained impairment in cardiac function (Table S1 in (Fischer et al., 2024)). There have been isolated reports of reversible issues with tamoxifen; by limiting our treatment to the pre‐MI period and treating all mice with tamoxifen, we limited the impact of this variable (i.e., both groups of mice received tamoxifen). Hence, we are confident with the strategy we have used, though a limitation is noted. The use of both sexes is a clear strength. Many studies continue to be published in only one sex (usually male). Such differences further underscore the value of including both sexes. Completing a study with both sexes requires twice the time, money, and resources, but is worth it because of the relevance to half the human population. Despite the value, this is often overlooked by journals and even grant review panels, which is unfortunate. Our present data indicate sex differences, yet we do not know why they occurred, which was not a goal of this study. Along with the strength of using both sexes, there are limitations to consider. The mice used in this study lacked any risk factors as would be expected in a corresponding human population suffering an MI. In addition, there was apparent attrition of recombined fibroblasts after MI. That is, despite the clear demonstration of a reduction in *Has2* expression at the beginning of the experiment, the difference was not apparent at the end of the protocol. It is possible that the increased severity of heart failure promoted excessive activation of non‐recombined fibroblasts. In addition, because we were not administering tamoxifen during/after MI, it is possible that non‐recombined fibroblasts outcompeted recombined (i.e., *Has2* ^*−/−*^). Hence, continuing tamoxifen treatment after MI (e.g., via chow) may enhance the deletion of fibroblasts after MI, that is, new post‐MI fibroblasts would also be recombined. Such design is being incorporated in other studies in the laboratory.

In conclusion, fibroblast‐derived hyaluronan supports the compensatory response early after MI in male mice. Future studies will address the impact of hyaluronan in ventricular remodeling after scar formation.

## AUTHOR CONTRIBUTIONS

**Danielle T. Little**: Designed and performed experiments; analyzed and interpreted data; provided funding; wrote and revised manuscript. **Kenneth R. Brittian**, **Caitlin Howard**, **Ning Chen**: Performed experiments; analyzed data. **Emma Pendergraft**, **Casey Colley**: Analyzed data. **Richa Singhal**, **Joseph B. Moore**: Analyzed data; revised manuscript. **Yu Yamaguchi**: Provided critical reagents. **Marcin Wysoczynski**: Designed experiments; revised manuscript. **Yibing Nong**: Performed experiments; revised manuscript. **Steven P. Jones**: Conceived project; designed experiments; interpreted data; provided funding; wrote and revised manuscript.

## FUNDING INFORMATION

DL was supported by an NIH Predoctoral Fellowship (F31 HL162518). CH was supported by an American Heart Association Predoctoral Fellowship (25PRE1410255). Grants from the NIH supported infrastructure/equipment in the Center (P30 GM127607, S10 OD038345, and R01 GM127495) and in the laboratory of SPJ (R01 HL163272). SPJ has also been funded by the American Heart Association and the Jewish Heritage Fund for Excellence (University of Louisville). We appreciate the in‐kind support from the Robley Rex VA Medical Center for providing the Nikon A1R MP multi‐photon microscope used in this study. The content is solely the responsibility of the authors and does not necessarily represent the official views of the National Institutes of Health or any other funding agency.

## CONFLICT OF INTEREST STATEMENT

None of the authors has any relevant conflicts to declare.

## ETHICS STATEMENT

Appropriate biosafety and animal use approvals were in place at the time of the experiments. The authors have no relevant conflicts of interest to disclose. Data underlying the results in this study can be made available upon reasonable request. When possible (e.g., in vivo studies; pathology; downstream analyses of cell culture studies), experimenters were blinded to group assignment. To the extent possible, all technical details are provided; however, the authors can provide further clarification, if needed. Artificial intelligence was not used in the design, interpretation, or writing.

## Supporting information


**Figure S1.** Flow cytometry gating strategy. A CD11b antibody was first used to separate lymphocytes (CD11b^−^) and non‐lymphocytes (CD11b^−^). Within the CD11b^−^ population CD3 and B220 expression was used to differentiate between T cells and B cells, respectively. Cells that were Cd11b^+^Ly6G^+^ were identified as neutrophils. Within the CD11b^+^Ly6G^−^ cells, CD115 expression was used to separate monocytes (CD115^+^) and dendritic cells (CD115^−^). In peripheral blood samples (example shown above) CD11b^+^Ly6G^−^SSC^High^ cells were identified as eosinophils.
**Figure S2.** Deletion of *Has2* causes an increase in *Has1* expression. (A) Relative mRNA expression of Has1 was significantly increased in fibroblasts isolated from *Has2* ^*−/−*^ mice compared to *Has2* ^*+/+*^ mice. (*n* = 4) (B) Relative mRNA expression of *Has3* do not significantly change in fibroblasts isolated from *Has2* ^*−/−*^ mice compared to control. (*n* = 4) Unpaired *t*‐test w/SEM.
**Figure S3.** Total LV hyaluronan accumulation is not reduced 7 days post‐MI in *Has2* deleted mice. (A) Representative images taken after staining with DAPI (blue), which stains for nuclei and HABP (red). (B) Quantification of tissue stained with hyaluronan binding protein shows no significant changes in hyaluronan accumulation in the total left ventricle in *Has2* ^*−/−*^ mice, 7 days post‐Ml, compared to *Has2* ^*+/+*^ mice. *Has2* ^*+/+*^ *n* = 16 (8F, 8M), *Has2* ^*−/−*^ *n* = 20 (12F, 8M). Unpaired *t*‐test with Mann–Whitney test. HA ELISA shows no significant differences in total HA in *Has2* ^*−/−*^ MI heart tissue (*n* = 8) compared to *Has2* ^*+/+*^ MI heart tissue (*n* = 9). Unpaired *t*‐test (C) Males. (D) Females.
**Figure S4.** *Has2* mRNA is not significantly decreased in *Has2* deleted fibroblast 7 days post‐MI. (A) Representative gel showing relative size and abundance of hyaluronan. High molecular weight hyaluronan (HA^HMW^) was loaded as a molecular weight marker/positive control. (B) Quantification of stained agarose gels. Deletion of *Has2* did not show sustained reduction of cardiac fibroblasts accumulated HA 7 days post‐MI *n* = 9 (5F, 4M) compared to *Has2* ^*+/+*^ cardiac fibroblasts 7 days post‐MI *n* = 9 (5F, 4M) Unpaired *t*‐test. Relative mRNA expression of *Has2*, *Has1* and *Has3* is not significantly changed in fibroblasts isolated from *Has2* ^*−/−*^ mice 7 days post‐MI compared to *Has2* ^*+/+*^ mice. *n* = 8 (5F, 3M) Unpaired *t*‐test w/SEM. (C) Males (D) Females.
**Table S1.** Antibodies and fluorochromes used for flow cytometry experiments.
**Table S2.** Gravimetric and echocardiographic data from *Has2* ^*fl/fl*^::Col1a2‐Cre 12–16 week old male and female mice. Mean ± SEM, *p < 0.05 when we compare 7 days post‐MI *Has2* ^+/+^ versus 7 days post‐MI *Has2* ^*−/−*^.
**Table S** Deletion of *Has2* in fibroblasts causes significant increase in T cells in female mice. Flow cytometry was used to characterize basic immune cell populations in the peripheral blood, bone marrow, and spleen of infarcted mice. Values are reported as the average number of cells ± the standard deviation. Text highlighted in red indicates a significant difference between the *Has2* ^*+/+*^ and *Has2* ^*−/−*^ groups (unpaired *t*‐test).
