# Supplementary material for: Fibroblast Has2 limits acute heart failure following myocardial infarction in male mice
Source: Physiol Rep. 2025 Nov 18;13(22):e70611. doi: 10.14814/phy2.70611 (PMC12624274; doi:10.14814/phy2.70611)
Supplement: Supplementary file 1 — Figure S1. Flow cytometry gating strategy. A CD11b antibody was first used to separate lymphocytes (CD11b−) and non‐lymphocytes (CD11b−). Within the CD11b− population CD3 and B220 expression was used to differentiate between T cells and B cells, respectively. Cells that were Cd11b+Ly6G+ were identified as neutrophils. Within the CD11b+Ly6G− cells, CD115 expression was used to separate monocytes (CD115+) and dendritic cells (CD115−). In peripheral blood samples (example shown above) CD11b+Ly6G−SSCHigh cells were identified as eosinophils. Figure S2. Deletion of Has2 causes an increase in Has1 expression. (A) Relative mRNA expression of Has1 was significantly increased in fibroblasts isolated from Has2 −/− mice compared to Has2 +/+ mice. (n = 4) (B) Relative mRNA expression of Has3 do not significantly change in fibroblasts isolated from Has2 −/− mice compared to control. (n = 4) Unpaired t‐test w/SEM. Figure S3. Total LV hyaluronan accumulation is not reduced 7 days post‐MI in Has2 deleted mice. (A) Representative images taken after staining with DAPI (blue), which stains for nuclei and HABP (red). (B) Quantification of tissue stained with hyaluronan binding protein shows no significant changes in hyaluronan accumulation in the total left ventricle in Has2 −/− mice, 7 days post‐Ml, compared to Has2 +/+ mice. Has2 +/+ n = 16 (8F, 8M), Has2 −/− n = 20 (12F, 8M). Unpaired t‐test with Mann–Whitney test. HA ELISA shows no significant differences in total HA in Has2 −/− MI heart tissue (n = 8) compared to Has2 +/+ MI heart tissue (n = 9). Unpaired t‐test (C) Males. (D) Females. Figure S4. Has2 mRNA is not significantly decreased in Has2 deleted fibroblast 7 days post‐MI. (A) Representative gel showing relative size and abundance of hyaluronan. High molecular weight hyaluronan (HAHMW) was loaded as a molecular weight marker/positive control. (B) Quantification of stained agarose gels. Deletion of Has2 did not show sustained reduction of cardiac fibroblasts accumulated HA 7 [file PHY2-13-e70611-s001.pdf]

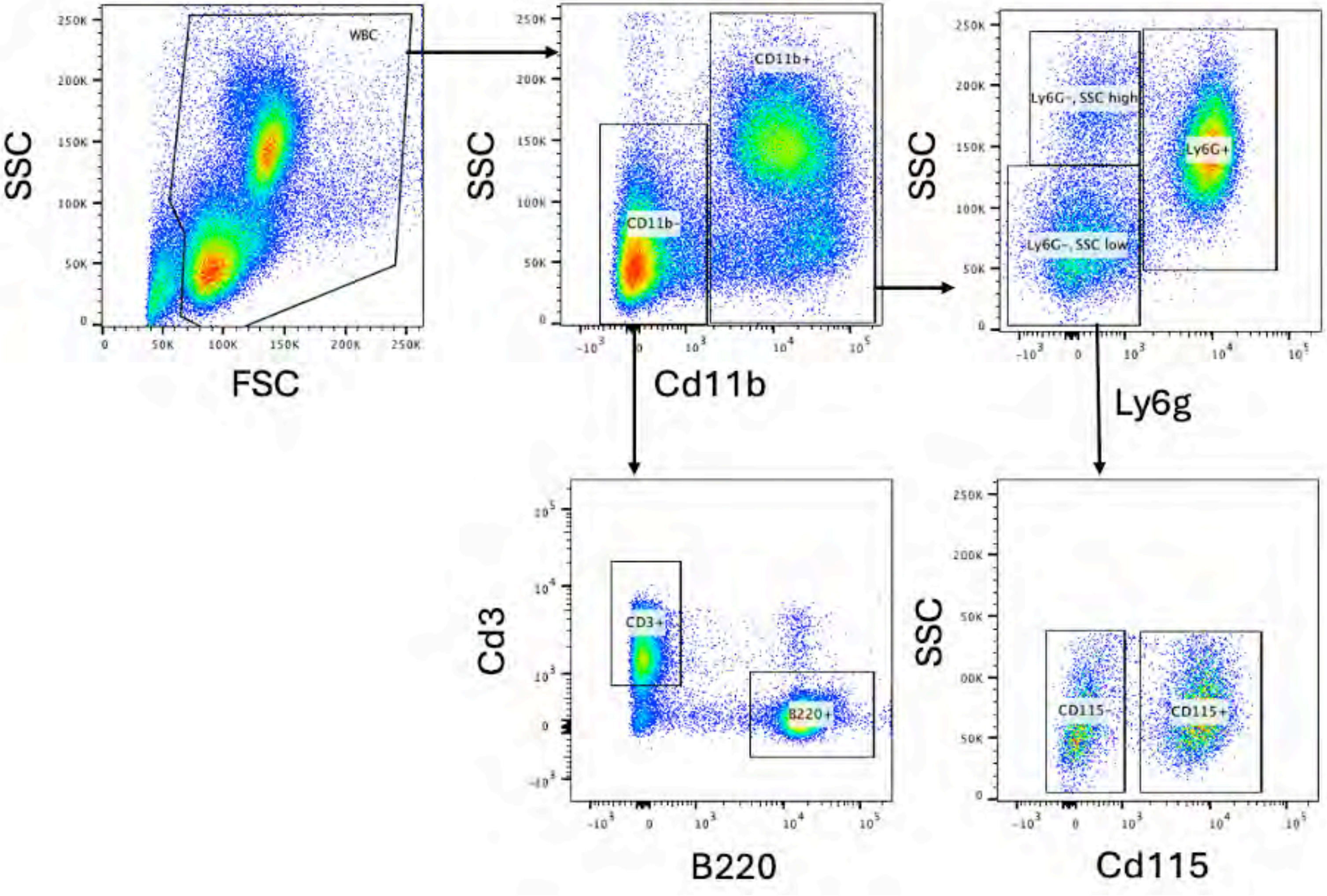

Supplemental Figure 1. *Flow cytometry gating strategy.* A CD11b antibody was first used to separate lymphocytes (CD11b^{-}) and non-lymphocytes (CD11b^{-}). Within the CD11b^{-} population CD3 and B220 expression was used to differentiate between T cells and B cells respectively. Cells that were CD11b^{+}Ly6G^{+} were identified as neutrophils. Within the CD11b^{+}Ly6G^{-} cells, CD115 expression was used to separate monocytes (CD115^{+}) and dendritic cells (CD115^{-}). In peripheral blood samples (example shown above) CD11b^{+}Ly6G^{-}SSC^{High} cells were identified as Eosinophils.

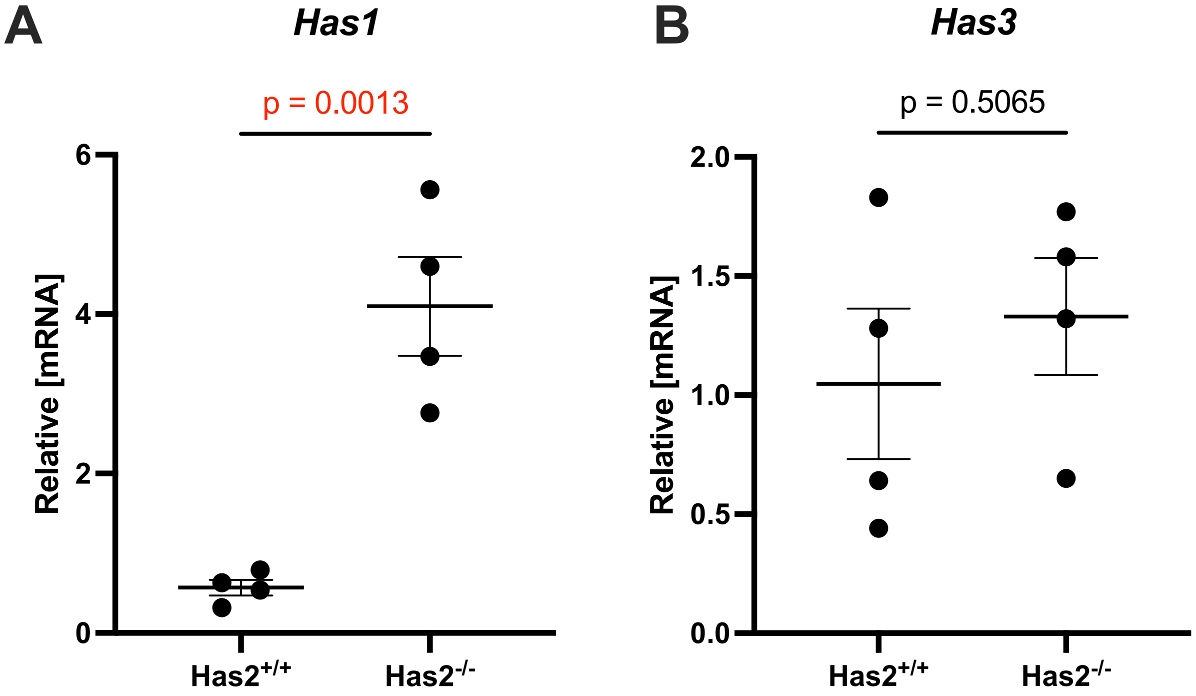

Supplemental Figure 2. *Deletion of Has2 causes an increase in Has1 expression.* (A) Relative mRNA expression of *Has1* was significantly increased in fibroblasts isolated from *Has2*$^{-/-}$ mice compared to *Has2*$^{+/+}$ mice. (n=4) (B) Relative mRNA expression of *Has3* does not significantly change in fibroblasts isolated from *Has2*$^{-/-}$ mice compared to control. (n=4) Unpaired t-test w/ SEM.

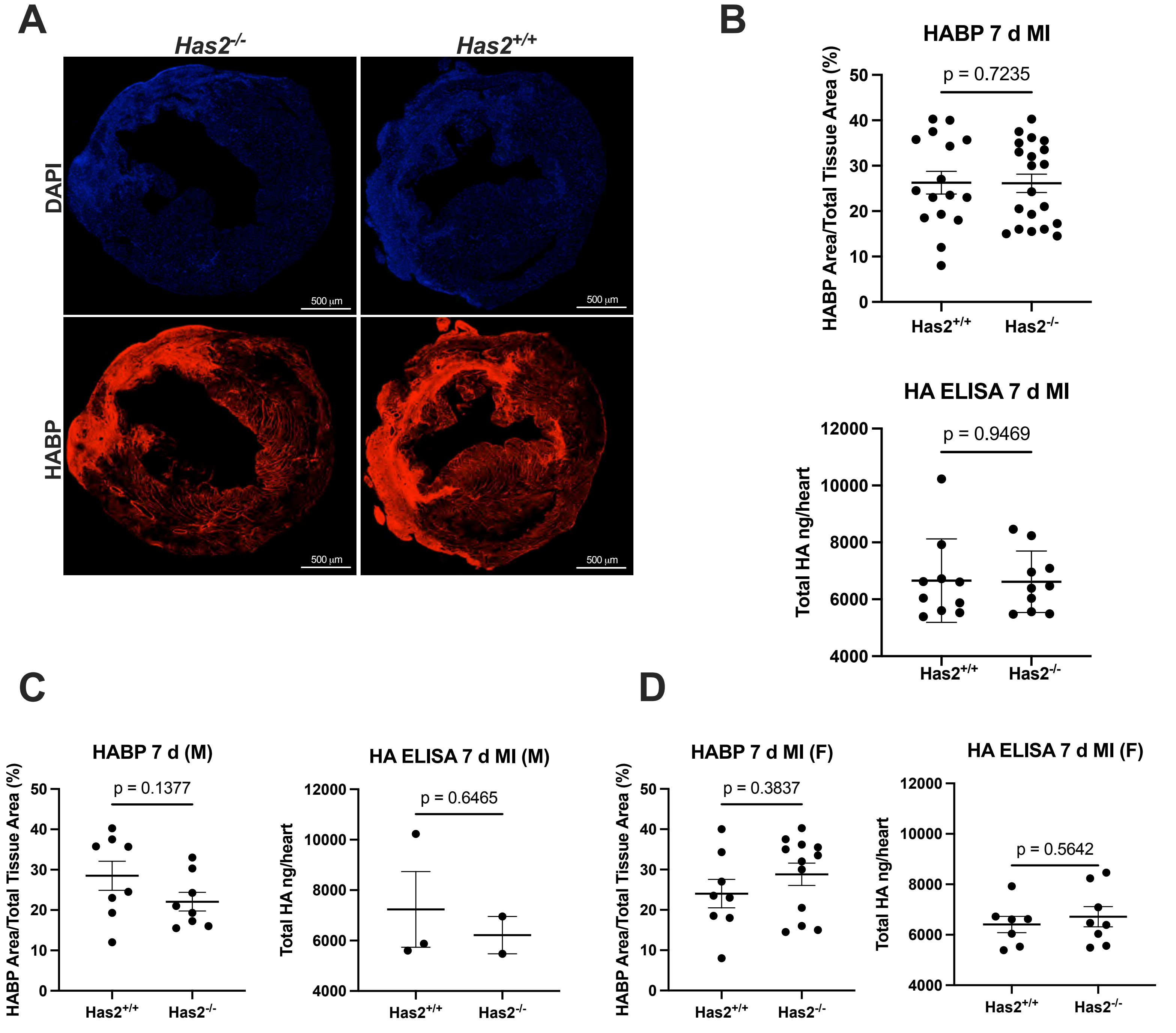

Supplemental Figure 3. *Total LV hyaluronan accumulation is not reduced 7 d post-MI in Has2 deleted mice.* (A) Representative images taken after staining with DAPI (blue), which stains for nuclei and HABP (red). (B) Quantification of tissue stained with hyaluronan binding protein shows no significant changes in hyaluronan accumulation in the total left ventricle in *Has2*$^{-/-}$ mice, 7 d post-MI, compared to *Has2*$^{+/+}$ mice. *Has2*$^{+/+}$ n=16 (8F, 8M), *Has2*$^{-/-}$ n=20 (12F, 8M). Unpaired t-test with Mann-Whitney test. HA ELISA shows no significant differences in total HA in *Has2*$^{-/-}$ MI heart tissue (n=8) compared to *Has2*$^{+/+}$ MI heart tissue (n=9). Unpaired t-test (C) Males. (D) Females.

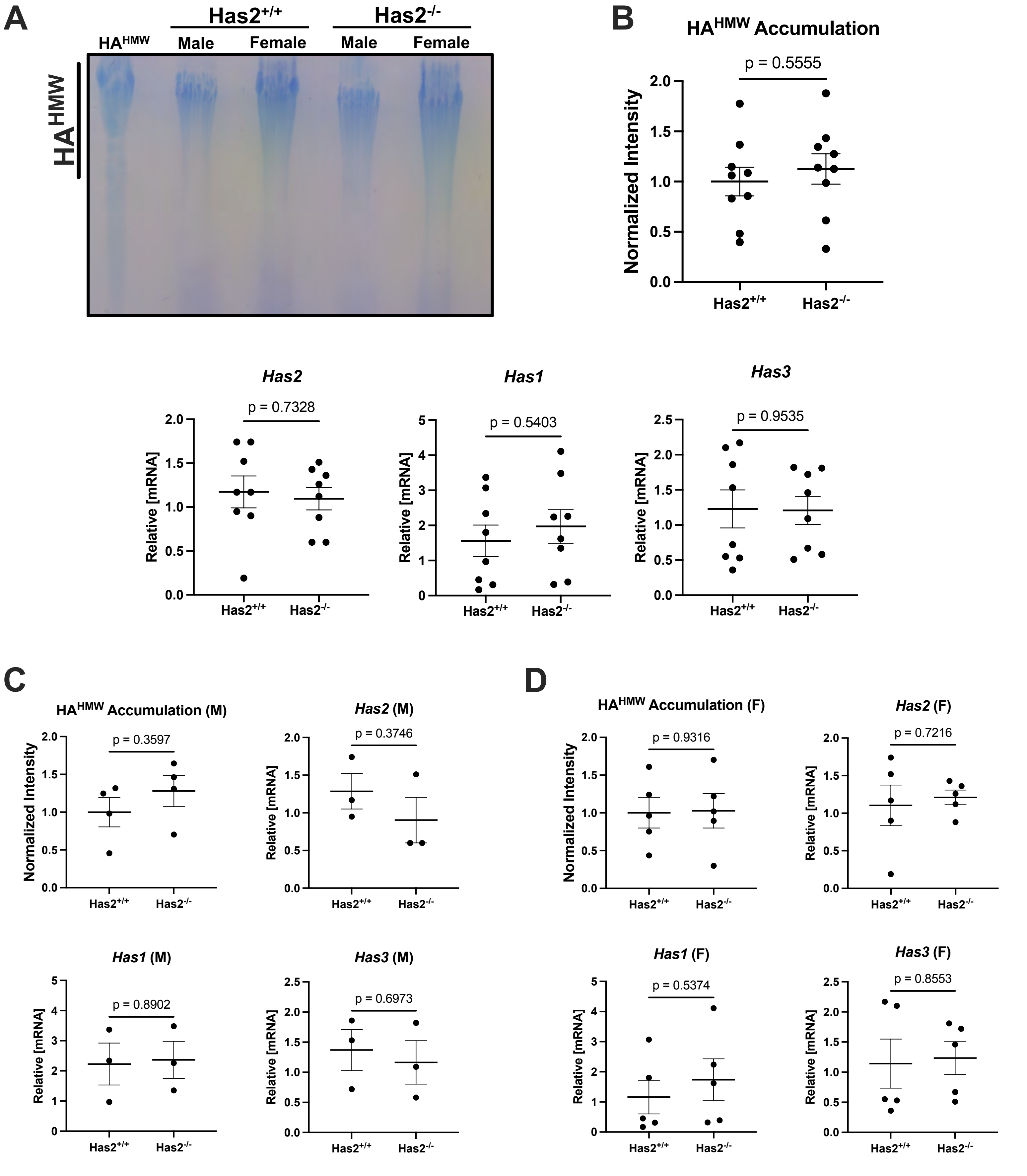

Supplemental Figure 4. *Has2 mRNA is not significantly decreased in Has2 deleted fibroblast 7d post-MI.* (A) Representative gel showing relative size and abundance of hyaluronan. High molecular weight hyaluronan (HA^{HMW}) was loaded as a molecular weight marker/positive control. (B) Quantification of stained agarose gels. Deletion of *Has2* did not show sustained reduction of cardiac fibroblasts accumulated HA 7 d post-MI n=9 (5F, 4M) compared to *Has2*$^{+/+}$ cardiac fibroblasts 7 d post-MI n=9 (5F, 4M) Unpaired t-test. Relative mRNA expression of *Has2, Has1* and *Has3* is not signifcantly changed in fibroblasts isolated from *Has2*$^{-/-}$ mice 7 d post-MI compared to *Has2*$^{+/+}$ mice. n=8 (5F, 3M) Unpaired t-test w/ SEM. (C) Males (D) Females

Target	Fluorochrome	Clone	Supplier	Inventory Number
CD11b	APC	M1/70	Invitrogen	17-0112-82
Ly6G	PE	1A8	Invitrogen	12-9668-82
B220	PE-Cy7	RA3-6B2	Invitrogen	25-0452-82
CD3	eF450	145-2C11	Invitrogen	48-0031-82
CD115	BV605	AFS98	Biolegend	135517

Supplemental Table 1. *Detailed flow cytometry materials.*

	All Sexes		Females		Males	
	7d MI $Has^{+/+}$	7d MI $Has^{-/-}$	7d MI $Has^{+/+}$	7d MI $Has^{-/-}$	7d MI $Has^{+/+}$	7d MI $Has^{-/-}$
**Body weight (g)**	23.9 ± 0.5	22.7 ± 0.4	22 ± 0.4	21.1 ± 0.3	25.8 ± 0.7	24.6 ± 0.4
**HR (bpm)**	581 ± 5	573 ± 6	582 ± 6	577 ± 7	581 ± 9	568 ± 9
**EF (%)**	21 ± 2	22 ± 2	20 ± 2	23 ± 2	23 ± 2	20 ± 3
**FS (%)**	11 ± 1	11 ± 1	10 ± 2	12 ± 2	12 ± 2	11 ± 2
**EDV (μL)**	141.1 ± 11	129 ± 1	116 ± 7	104 ± 7	166 ± 18	158 ± 17
**ESV (μL)**	115 ± 11	105 ± 10	95 ± 8	83 ± 8	134 ± 19	131 ± 18
**IVRT (ms)**	17 ± 1	18 ± 0.4*	17 ± 1	18 ± 1	17 ± 1	18 ± 1
**LVIDd (mm)**	5.1 ± 0.2	4.9 ± 0.2	4.7 ± 0.1	4.5 ± 0.1	5.4 ± 0.3	5.3 ± 0.3
**LVIDs (mm)**	4.6 ± 0.2	4.4 ± 0.2	4.3 ± 0.2	4.0 ± 0.2	4.9 ± 0.3	4.7 ± 0.3
**LVPWd (mm)**	0.8 ± 0.1	0.8 ± 0.1	0.8 ± 0.1	0.8 ± 0.1	0.8 ± 0.1	0.8 ± 0.1
**LVPWs (mm)**	1.0 ± 0.1	0.9 ± 0.1	0.9 ± 0.1	0.9 ± 0.1	1.0 ± 0.1	0.9 ± 0.1
**LVAWd (mm)**	0.6 ± 0.0	0.6 ± 0.0	0.6 ± 0.1	0.7 ± 0.1	0.6 ± 0.1	0.5 ± 0.0
**LVAWs (mm)**	0.7 ± 0.1	0.7 ± 0.1	0.7 ± 0.1	0.8 ± 0.1	0.7 ± 0.1	0.5 ± 0.0
**"n"**	34	37	17	20	17	17

Supplemental Table 2: *Gravimetric and echocardiographic data from* $Has2^{fl/fl}$::Col1a2-Cre *12–16 wk old male and female mice.* Mean ± SEM, *p<0.05 when we compare 7 d post-MI $Has2^{+/+}$ vs 7 d post-MI $Has2^{-/-}$.

Peripheral Blood Cells/µL				
	Male		Female	
	WT (n=14)	$Has2^{-/-}$ (n=10)	WT (n=15)	$Has2^{-/-}$ (n=21)
Eosinophils	480 ± 499	396 ± 280	187 ± 157	305 ± 330
Neutrophils	13133 ± 5302	14637 ± 9056	6634 ± 2073	9117 ± 4368
Monocytes	2214 ± 1882	1628 ± 1181	629 ± 376	998 ± 692
Dendritic Cells	1000 ± 555	897 ± 351	576 ± 250	789 ± 428
T cells	3666 ± 1875	3244 ± 1051	1762 ± 791	2890 ± 1352
B cells	4877 ± 2990	4328 ± 2506	2042 ± 990	3421 ± 2904
**Bone Marrow Cells/Tibia**				
	Male		Female	
	WT (n=15)	$Has2^{-/-}$ (n=16)	WT (n=16)	$Has2^{-/-}$ (n=22)
Neutrophils	$3.7 \times 10^6 \pm 1.2 \times 10^6$	$3.5 \times 10^6 \pm 9.6 \times 10^5$	$2.6 \times 10^6 \pm 5.2 \times 10^5$	$2.3 \times 10^6 \pm 6.5 \times 10^5$
Monocytes	$5.3 \times 10^5 \pm 2.2 \times 10^5$	$5.5 \times 10^5 \pm 2.1 \times 10^5$	$3.4 \times 10^5 \pm 1.1 \times 10^5$	$3.3 \times 10^5 \pm 1.2 \times 10^5$
Dendritic Cells	$2.6 \times 10^5 \pm 1.2 \times 10^5$	$2.6 \times 10^5 \pm 2.1 \times 10^5$	$1.9 \times 10^5 \pm 5.7 \times 10^4$	$2.2 \times 10^5 \pm 7.1 \times 10^4$
T cells	$8.9 \times 10^4 \pm 6.02 \times 10^4$	$8.6 \times 10^4 \pm 2.7 \times 10^4$	$7.9 \times 10^4 \pm 2.1 \times 10^4$	$8.2 \times 10^4 \pm 2.6 \times 10^4$
B cells	$2.9 \times 10^5 \pm 2.3 \times 10^5$	$2.2 \times 10^5 \pm 8.4 \times 10^4$	$2.2 \times 10^5 \pm 8.5 \times 10^4$	$2.2 \times 10^5 \pm 8.8 \times 10^4$
**Spleen Cells/mg Tissue**				
	Male		Female	
	WT (n=10)	$Has2^{-/-}$ (n=7)	WT (n=11)	$Has2^{-/-}$ (n=12)
Neutrophils	10189 ± 8502	10748 ± 5502	10219 ± 10090	11248 ± 6644
Monocytes	2224 ± 2547	2598 ± 2062	1978 ± 2352	2683 ± 2145
Dendritic Cells	2786 ± 1815	3249 ± 1422	3313 ± 3119	4574 ± 2887
T cells	16922 ± 7794	23592 ± 8522	18286 ± 10875	26701 ± 16813
B cells	10242 ± 6706	19254 ± 11823	9241 ± 9193	17098 ± 16917

Supplemental Table 3. *Deletion of Has2 in fibroblasts causes significant increase in neutrophils and T cells in female mice.* Flow cytometry was used to characterize basic immune cell populations in the peripheral blood, bone marrow, and spleen of infarcted mice. Values are reported as the average number of cells ± the standard deviation. Text highlighted in red indicates a significant difference between the *$Has2^{+/+}$* and *$Has2^{-/-}$* groups (unpaired t-test).
